# Hammering nails with a screwdriver: how GPs perceive video consultations

**DOI:** 10.3399/BJGPO.2024.0010

**Published:** 2024-10-30

**Authors:** Magnus Repstad Wanderås, Eirik Abildsnes, Elin Thygesen, Santiago Gil Martinez

**Affiliations:** 1 Faculty of Health and Sport Sciences, University of Agder, Grimstad, Norway; 2 Institute of Health and Society, Faculty of Medicine, University of Oslo, Oslo, Norway

**Keywords:** general practice, remote consultation, telemedicine, COVID-19

## Abstract

**Background:**

Early in the COVID-19 pandemic, the use of video consultation (VC) expanded considerably, with GPs indicating high satisfaction with it. However, use of VC declined as lockdown measures were eased.

**Aim:**

To explore reasons why VC use has declined in Norwegian general practice since the start of the pandemic by investigating GPs’ experiences with VC and their attitudes towards it in a post-pandemic setting.

**Design & setting:**

Qualitative study using semi-structured interviews with 13 GPs in southern Norway between May 2022 and March 2023.

**Method:**

Data analysis was conducted by applying the six steps of Braun and Clarke’s reflexive thematic analysis.

**Results:**

Although the implementation of VCs was unplanned, most participants were able to use this modality without much problem. Several GPs initially envisioned long-term VC use. However, despite certain positives, VCs were largely sidelined in favour of face-to-face and telephone consultations, owing to their practicality and VC’s limited usefulness when considering the extra effort required. Nonetheless, GPs recognised ways of using VC that might exploit its strengths, but they highlighted how its sustained use would require them to replace other consultation modalities. They also identified extrinsic factors that might lead to the increased use of VC, including improved VC technology and patient demand.

**Conclusion:**

Although VC is now part of many GPs’ consultation toolboxes, its perceived relative lack of usefulness and extra effort compared with other remote consultation modalities mean that most GPs have chosen to abandon it as a routine consultation modality.

## How this fits in

Previous studies showing the promise of video consultation (VC) in general practice were mostly conducted with limited scope or early in the COVID-19 pandemic. This study adds a more longitudinal understanding of reasons why VC has been largely abandoned by Norwegian GPs. We discuss the implementation of VC in light of theory and identify areas for future research on remote consultations in general practice.

## Introduction

Over the past 10 years, interest in VC as an innovation and consultation modality in health care has been growing, with studies showing how it can be acceptable, safe, and effective in selected patient groups.^1,2^ A scoping review of experiences with VC in primary care, published in 2020, found a similar result in the general practice setting.^3^ Despite this, the use of VC in general practice was low before the COVID-19 pandemic.^4–8^

The pandemic has been characterised as a *‘natural experiment’* and an *‘opportunity in a crisis’*, pertaining to the uptake of VC in health care.^9,10^ The use of remote consultations, including VC, increased in primary health care in several countries during the pandemic, with follow-up and simpler issues more suitable than potentially serious illness in need of physical examination.^11^ During the first COVID-19 lockdown, almost 90% of GP offices in Norway could offer VC.^12^ Based on their initial experiences, Norwegian GPs envisioned substantial long-term use of VC.^6^

However, even during the early months of the pandemic, there was a decline in VCs among GPs in Norway (The Norwegian Directorate of E-health, unpublished data, 2020). The numbers during and after the pandemic indicate the continuing struggle for VC to become a mainstay consultation modality,^8,13,14^ illustrating a discrepancy between 1) positive pre-pandemic research findings and positive experiences early in the pandemic, and 2) the current low use of VC. Therefore, this study aims to explore why VC use has declined in Norwegian general practice by investigating GPs’ experiences with it since the start of the pandemic and their attitudes towards it in a post-pandemic setting.

## Method

### Design

This is a qualitative study underpinned by a critical realist ontological understanding.^15^ This allows for an experiential-oriented and a reflexive approach. As a trained physician and former substitute GP, the first author (MRW) has experienced working with VC in general practice. MRW’s experiences are an integral part of their situatedness as a researcher and an important part of the lens through which they collected and interpreted data during the analysis. The study was conducted with an inductive approach, with the analysis being *‘grounded in the data’*.^16^ The research team sought to achieve methodological integrity through alignment and coherence between the research design, research questions, and theoretical assumptions.^17^ The Standards for Reporting Qualitative Research^18^ guided the reporting.

In the discussion, we invoked elements from Rogers’ diffusion of innovations theory^19^ to inform further insight from the results. We chose this theory owing to the structure of Norwegian general practice, where each GP is free to choose which consultation modalities they want to, and where choice of modalities can differ within GP offices. This individuality in modality choice warranted use of the diffusion of innovations theory to further examine possible reasons for individual adoption or non-adoption of VC. We also invoked Gkeredakis and colleagues’ three perspectives on crisis and digital change^20^ for further insight relating to the special circumstances of the uptake in VC use.

### Participants

The participants were GPs working in Norway. There were three inclusion criteria. First, they had to be GP specialists or have started their GP specialisation, since this ensured a minimum knowledge of the GP role. Second, they had to work at standard GP offices in Norway, thereby excluding physicians working as primary care physicians in private healthcare clinics. Third, they had to have used VCs at some point in their GP careers, since the study sought to explore experiences with VCs.

In total, 13 participants from southern Norway were included in the study (Table 1). The number of participants was guided by the principle of information power.^21^ The participants had been recruited in a pragmatic manner through a professional GP network and represented a wide variation in age, sex, years of experience in general practice, and use of VC. Eight were acquaintances of MRW, who had made direct contact with them via Facebook Messenger or text message. The last five participants were recruited indirectly through contact via text message or email.

**Table 1.** Overview of the participants

Participant	Age, years	Sex	Years as GP	Specialist or not	Use of VC at time of interview
GP 1	31	Male	2.5	No	Very low
GP 2	33	Male	3	No	Very low
GP 3	36	Female	6	No	Very low
GP 4	65	Male	26	Yes	Actively using VC
GP 5	52	Female	25	Yes	Very low
GP 6	50	Female	12	Yes	Very low
GP 7	36	Male	5	No	Very low
GP 8	44	Male	10	Yes	Sometimes
GP 9	63	Male	31	Yes	Very low
GP 10	47	Male	10	Yes	Very low
GP 11	48	Female	21	Yes	Actively using VC
GP 12	40	Female	5	No	Actively using VC
GP 13	45	Female	11.5	Yes	Very low

VC = video consultation.

### Data collection

Data were collected through individual semi-structured interviews supported by an interview guide with questions addressing aspects related to the research aims (see Supplementary Information S1). The interview guide was trialled in a pilot interview with a GP who matched the inclusion criteria. Throughout the data collection process, the interview guide was adjusted to reflect relevant topics that had emerged during interviews.

A total of 13 interviews were conducted by MRW between May 2022 and March 2023. Each interview was conducted in-person and lasted 40–60 minutes. The interviews were audio-recorded and transcribed verbatim by MRW. MRW also recorded and transcribed reflection notes after each interview, which were then included in the data analysis.

### Data analysis

The analysis was conducted by applying the six steps of Braun and Clarke’s method of reflexive thematic analysis,^17^ which is an iterative process promoting a dynamic flow between the analytic steps. MRW was responsible for advancing the analytic process, allowing engagement with the data in a reflexive manner. The research team contributed insights through their review of the initial coding, thematic structure and analytic drafts, and assessment of the analysis against critical questioning.

The first step of analysis consisted of data familiarisation, which was conducted by MRW by actively engaging in the interviews, recording post-interview reflection notes, transcribing interviews, and re-reading transcribed interviews before coding. The second step involved the systematic coding of the collected data. The data were coded twice using NVivo (version 12), a software solution for qualitative data analysis. The code labels were reviewed, and codes were merged when suitable. The third step consisted of generating initial themes from the codes. Initial thoughts were gathered and shaped into a preliminary theme structure, aiming for themes with shared meaning as opposed to topic summary themes.^22^ The fourth step consisted of developing and reviewing themes. Central organising concepts of the themes were identified, and theme definitions were written to evaluate the themes. Step five consisted of refining, defining, and naming themes and was conducted by the team. Step six involved writing the analytical text and the overall report, which was done by MRW, with the research team reviewing the report.

## Results

The thematic analysis resulted in the following three main themes: tooling up; hammering nails with a screwdriver; and a tool for the toolbox. The themes shared the concept of VC as a consultation tool.

### Tooling up

Before the pandemic, most participants had no experience with VCs. COVID-19 forced a rapid change in how GPs maintained contact with patients, and many turned to VC as a tool for conducting consultations. VCs were often adopted within days since it allowed contact with patients while minimising COVID-19 infection risk. Several participants experienced adopting VC somewhat involuntarily:

*‘It was at a time of war, and there was this sense of necessity for the opportunity to see patients, and that’s why we did it.’* (GP 8)

The GPs did not receive formal VC training, but several were shown by colleagues how to use the modality. A couple of the participants highlighted this lack of lengthy training in some sense to be a hallmark of practising medicine, since physicians must often learn on the spot:

*‘I was shown* [how to use VC] *by a colleague. That was it. Kind of like "see one, do one, teach one" — isn’t that what we do in medicine?’* (GP 3)

None of the participants’ offices developed guidelines for VC use. This led to some participants struggling with the technology and feeling uncertain about VC. Contrarily, many participants were initially rather positive and expressed gratitude for meeting patients at all in an uncertain period. They found VC to be easy to use and envisioned using them longer than they did:

*‘Most* [GPs in the office] *thought it was exciting and fun in the beginning, being able to do something new.’* (GP 5)

Two participants reflected on how the pandemic-forced implementation affected uptake. One participant thought that VC use would have been much lower without the semi-forced implementation, while the other participant discussed how lack of a more traditional implementation process might have impaired long-term use:

*‘It went from nothing to — bang — everything. If it had been phased in, been more controlled ... then it probably would have been very different. I think so.’* (GP 1)

### Hammering nails with a screwdriver

The participants extensively reflected on the perceived gains and losses through the use of VC. Looking at VC use alone, they highlighted several positives. VCs allowed for flexible workdays, allowing GPs to work from outside the office. They also made it easier to follow-up patients living far away. The value of VCs increased when the GPs knew the patients beforehand:

*‘If I am to succeed in using video to assess a patient’s general health condition ... that requires me to know how that patient’s health condition normally is.’* (GP 4)

The perceived gains and losses from VCs became more nuanced when their use was viewed opposite other consultation modalities. Among the participants and their colleagues, VCs were phased out as patients were allowed back into offices after lockdown:

*‘You usually don’t use a screwdriver to hammer a nail. There is something about the right tool at the right time, and that when infection prevention wasn’t the biggest concern any longer, you would rather go back to what is more traditional.’* (GP 2)

Participants reflected widely on the gains and losses of VC use compared with face-to-face consultations. The benefits of VCs included such consultations usually being shorter and often with fewer issues raised. This time-saving benefit was mentioned by several participants.

Many spoke of VCs and the GP–patient relationship. Most found it challenging to establish and maintain GP–patient relationships via VC. They were sceptical about relationships established and maintained exclusively via video. Quality of communication was also highlighted, where face-to-face consultations, with their non-verbal elements, were viewed as superior to VCs. One participant explained how they found communication to be of integral importance in general practice:

*‘I know I am going to sound old-fashioned, but ... because* [face-to-face] *communication really surpasses all our tools and ways of examination, I believe that communication is better* [when the patient is at the office].*’* (GP 4)

Most participants agreed that VCs could supplement, but not replace, the still superior face-to-face consultations, and highlighted the need for GPs to be aware of the continuing need for physical meetings, as VCs fall short in relational, emotional, and *‘ritual’* aspects:

*‘There are certain almost “rituals” or “ritual actions” that GPs do ... One can say a lot about the specificity and sensitivity of a stethoscope examination, but it has a major effect in making the patient feel they are being taken seriously.’* (GP 2)

Participants also spoke about gains and losses between VCs and other forms of remote consultation. They mostly stated the differences between VCs and telephone consultations (TCs) in this study, highlighting several benefits of VCs compared with TCs. Several participants viewed VCs as superior to TCs in terms of communication, enabling richer GP–patient relationships:

*‘I think it is easier to relate to someone you can see and interpret in a broader sense than a voice on the telephone.’* (GP 2)

Another benefit of VCs is how the visual element provides extra information, allowing richer assessments, particularly when assessing children. However, several participants regarded the extra visual information obtained from a VC, compared with a TC, as not necessarily important:

*‘A great deal of issues aren’t suitable for remote consultations, and the rest of it might as well be handled via telephone.’* (GP 8)

Besides this, participants highlighted several other reasons why they often ended up using TCs instead of VCs. They mentioned how they were unsure of when a VC would be the best consultation modality among the options at hand. Compared with VCs, TCs also demanded more effort from the GPs and reduced the possibility for multi-tasking. They also mentioned how they had started using more TCs instead of VCs after they were allowed to take payment and receive reimbursement for a TC:

*‘*[…] *If that hadn’t happened, then I would have had to continue with VCs, as I wouldn’t be getting paid for the consultations otherwise.’* (GP 13)

The most prominent reason for choosing a TC over a VC, shared by almost all participants, was the simple fact that in a hectic workday, GPs would make this choice owing to the relative speed and ease of a TC:

*‘Because all my colleagues have stopped using VCs, I have also realised that it’s faster with a telephone. And in a* [setting] *where time is a valuable resource, I can maybe just take a telephone, rather than a VC*, *where issues are established and clarified.’* (GP 8)

### A tool for the toolbox

Even though VC use declined among participants and most opted out of using VCs on a regular basis, they still believed that VC had settled as a consultation modality option in the ‘GP toolbox’. They argued that GPs should try to be clear on what VCs are suitable and unsuitable for, and how physical meetings should still be prioritised:

*‘I think* [VC] *is a tool that may be fine to use from time to time. And I think the most important thing is to see the patient — be able to touch the patient*.*’* (GP 7)

Several participants spoke of a potential paradoxical situation. GPs rarely wanted to replace face-to-face meetings with VCs; however, they stressed how the sustained use of VCs would have them substitute face-to-face consultations. The alternative would be having VCs prioritised over every other consultation method:

*‘*[VCs] *need to replace rather than be in addition to* [face-to-face consultations]*. If you sit at night having a VC in addition to regular consultations, that might be troubling.’* (GP 2)

Participants reflected on possible cases whereby having VCs on hand in general practice would be useful going forward. Considering the recent pandemic, some participants emphasised VCs’ proficiency in emergency preparedness, maintaining consultation activity if a new crisis occurs. Participants also illustrated use of VC as part of a *‘hybrid consultation series’* with alternate video and face-to-face consultations, and also how VCs can be useful in triaging urgency of issues.

Participants identified several factors that might drive them towards increased use of VCs, none of which GPs can control themselves. Most agreed that more intuitive VC technology would likely lead to increased use. Another factor is patient preference, with participants observing patients preferring or even expecting VCs as an option, and that GPs ought to adapt to this change in demand. Some participants reflected on how the pandemic had made healthcare personnel and patients more comfortable with online communication and how this might have influenced patients’ consultation preferences. Participants also mentioned how demand for VC might increase further going forward as digitally competent patients get older:

*‘Of course, there is a new generation now in their 50s and 60s who are used to digital solutions. I think that with them, I could get some more of those patients through VCs.’* (GP 3)

One last factor that participants reflected on was competition from private healthcare companies outside the GP scheme, some of which offer VCs as part of their businesses and others exclusively deliver VCs. While a couple of participants were not driven to change their practice owing to this competition, more accepted it and found VCs to perhaps be necessary to ‘keep up’:

*‘If the patients’ expectations are for the GP to be available via VC because* [private healthcare companies] *are available via video, then I would think that we should not sideline ourselves and say that we won’t bother because we are against* [private healthcare companies]*.’* (GP 8)

## Discussion

### Summary

This qualitative interview study explored Norwegian GPs’ experiences with and attitudes towards VCs in general practice, aiming to understand the reasons for their declining use of VCs. Although the implementation of VCs had been unplanned, most participants were able to use them without facing many problems. However, despite certain positives, they mostly went on to sideline VCs in favour of face-to-face and telephone consultations. Although the exploitation of VCs’ strengths could be improved, the participants highlighted how sustained use of VCs had required them to replace other consultations. Extrinsic factors, including improved VC technology and patient demands, might advance VC use.

### Strengths and limitations

This study benefited from data obtained from a group of participants who varied across age, sex, and years of clinical experience in general practice. Another strength, and one that separates this study from previous articles on the topic, is the longitudinal aspect of adoption, non-adoption, and abandonment that this study captures. The first author’s background in general practice enabled them to connect with participants on a professional level during the interviews, demonstrating the value of being an ‘insider researcher’ — that is, being part of the group studied^17^ — although it is possible that answers given to an ‘outsider researcher’ might also have helped shed light on other sides of the topic. The method of analysis allowed for researcher situatedness by permitting reflexivity during the process, understanding situatedness as a strength, and avoiding ‘positivism creep’ (positivist assumptions unknowingly slipping into reflexive thematic analysis, such as through concerns over researcher bias).^23^

Although the qualitative design of the study limited the generalisability of the findings, the clearly contextualised results were of a nature that should have relevance beyond the contexts of the study, promoting their transferability.^17^ While this study only investigated GPs’ perspectives of declining VC use, the topic would have been addressed more thoroughly if patient perspectives had been included. The study would also have been strengthened if viewpoints from support staff at general practices had been included. VC is a broad term encompassing several ways of interaction between doctor and patient, whereas most VCs in Norwegian general practice are either pre-planned, time-slotted appointments, or used as a substitute for TCs whenever available during the workday. A limitation of the study is that GPs’ experiences in this study are not distinguishable based on mode of VC use. Another potential limitation of the study is how the interviews took place a while after the start of the pandemic, meaning that for most of the participants, their extensive use of VC had been in the past, enabling the risk of recall bias. Although the choice of theory in this article was justified, the use of a more organisationally focused theory would likely have added further strength to the theoretical insights gained from the results of the study.

### Comparison with existing literature

Theory-informed approaches to implementation are integral for implementing eHealth in clinical care.^24^ In their diffusion of innovations theory,^19^ Rogers discussed four main elements of the diffusion of innovations. One of these elements is particularly relevant to this study because it pertains to the attributes of the innovation itself. Two attributes were highlighted as particularly important in the adoption of innovations, that is, relative advantage (whether an innovation is better than what it superseded and capturing subjective perceptions of advantage) and compatibility (whether an innovation is perceived as consistent with the existing values, experiences, and needs of its adopters). In this study, it became evident that the GPs found little relative advantage in VCs, and because VCs did not answer a particular need, they struggled to identify a clear ‘gap’ in their work that this modality was best suited to fill. Participants also highlighted communicative troubles and the loss of physical presence, which is perceived as important for conducting quality general practice. Thus, the relative advantage and compatibility of VCs in general practice seemed to be low.

The notion of COVID-19 as *‘an opportunity in a crisis’* warrants exploration of VC uptake in general practice through Gkeredakis and colleagues’^20^ three perspectives on crisis and digital change, namely, crisis as opportunity, disruption, and exposure. Crisis as opportunity allows the acceleration of innovation processes as well as the fermentation of new organisational paradigms to be enabled. This accelerated innovation was clearly manifested in the uptake of VCs in general practice, and it forced GPs to reflect on their place in the organisation of general practice.

Gkeredakis and colleagues’ second perspective, crisis as disruption, is contextualised within health care as describing how *‘elements of existing professional practices and ... occupational meanings* [were reshuffled]*, for example, what it means to examine a patient, perform diagnosis, monitor patients’ progress, etc.’* ^20^ Ideas consistent with these were illustrated in this study, such as the notion of hybrid consultation series or easier follow-up of patients who lived far away. However, most participants were unwilling to waive face-to-face consultations, and thus, the degree of disruption within general practice consultation practices can be questioned.

Gkeredakis and colleagues’ third perspective, crisis as exposure, highlights the significance, actions, and issues of people, social systems and groups, organisations, and infrastructure that were not necessarily noticed previously. One such exposure was towards the fact that GPs had largely been left to their own devices in setting up a remote consultation service, and although national efforts had been made to support healthcare personnel with video use,^25^ GPs were still left without formal training and clear guidelines for its implementation. Another exposure was how the pandemic accentuated the possibility of digital health inequality. The mention by participants of an upcoming generation of digitally competent older patients implies how today’s older people might be affected by a degree of digital health inequality if the use of digital consultations increases.

In their study from 2022, Greenhalgh and colleagues^13^ investigated why VC was largely either never adopted or soon abandoned in UK general practice post-pandemic. An important difference between their study and ours lies in how the NHS is a driver for remote-first consultations, while this is not the case with Norwegian healthcare governance. Still, there is high accordance between the studies. As in our study, GPs in the UK were still unsure of the relative advantage of VC, while the need to manage practice workload influenced the type of consultation offered. UK-based GPs also found the GP–patient relationship to be more easily initiated and maintained in face-to-face consultations. Interestingly, some GPs had been introduced to simpler and more intuitive VC software, but still, both clinicians and patients found the telephone more reliable and quicker. Although participants in the present study expressed how an improved VC solution might increase use, this suggests that a substantial improvement in VC technology might be necessary for the use of VC to increase. Despite the similarities in reasons for abandonment of VC between Greenhalgh and colleagues’ UK-based study and our study from Norway, experiences with VC will ultimately vary depending on the context and means of VC delivery, meaning that other reasons for both abandonment and sustained use of VC in primary health care can probably be found in other healthcare systems.

Norwegian GPs’ experiences with VC were investigated in a recent qualitative study.^26^ Several findings from that study are in line with the ones presented in this article, including how VCs may be more informative with previously known patients and how it can be useful for triage. That study also identified how long-term VC use might undermine relational trust, in line with our findings on the GP–patient relationship. However, the novelty of VC during the data collection, which took place in the early months of the pandemic, impaired the study from addressing possible reasons for declining VC use, which separates it from this article where the longitudinal aspect of the collected data formed an invaluable foundation for the presented findings.

### Implications for research and practice

This study has illustrated GPs’ scepticism towards VC and the currently bleak outlook for it as an important consultation modality. Providing VC is mandatory in the UK and will be mandatory by the end of 2024 in Denmark,^8,27^ despite VCs making up only 1.2% of all consultations in Danish general practice as of August 2022.^27^ Assing Hvidt and colleagues^8^ argued in a recent viewpoint on the low adoption of VC in post-COVID-19 Northern Europe that there is a misalignment in some countries between policy-level enthusiasm for digitalisation and the conditions needed for its implementation in the clinical context. Although there is no current discussion of whether to impose the mandatory provision of VC in Norwegian general practice, this study raises several concerns that need to be addressed if such a discussion emerges in Norway. Furthermore, future research should aim to understand the facilitators for the successful adoption of VC in general practices where this has occurred, and to examine differences in determinants for adoption of VC in general practice versus secondary healthcare services. This will further inform the differences between the organisational levels and the need for targeted implementation strategies that take organisational factors into account.

Future research should aim to understand more about GPs’ thoughts on how to conduct ‘good general practice’ with continuity and quality of care in a general practice where digital consultations and digital interfaces are likely to be more prominent. This topic would benefit from qualitative research that strives for more in-depth analysis. Research should also aim to capture if and how quality of care, continuity, and digital health inequality will evolve in an increasingly digital general practice. A recent study investigating training in remote consultation services revealed how learning occurs in the context of high workload, and while stressed staff often lost both motivation for and receptivity to training, the desire for further training was almost universal.^28^ These findings underscore the importance of further research on how to empower physicians to operate remotely. Future research should also aim to explore patient preferences and needs in an increasingly digital general practice, and how competition from private healthcare companies affects continuity and quality of care in general practice.
